# Medical undergraduates’ use of behaviour change talk: the example of facilitating weight management

**DOI:** 10.1186/1472-6920-13-7

**Published:** 2013-01-24

**Authors:** Sarah Peters, Louisa Bird, Hamaira Ashraf, Sehar Ahmed, Philip McNamee, Cassandra Ng, Jo Hart

**Affiliations:** 1 Manchester Centre for Health Psychology, School of Psychological Sciences, University of Manchester, Manchester, UK; 2 North Western Deanery, Greater Manchester, UK; 3 Medical School, University of Manchester, Manchester, UK; 4 UHSM Academy, Education and Research Centre, University Hospital of South Manchester, Southmoor Rd, Wyhenshawe Hospital, Manchester, M23 9LT, UK

**Keywords:** Behaviour change, Obesity, Undergraduate

## Abstract

**Background:**

Obesity, an increasing problem worldwide, is a leading cause of morbidity and mortality. Management principally requires lifestyle (i.e. behavioural) changes. An evidence-base exists of behaviour change techniques for weight loss; however, in routine practice doctors are often unsure about effective treatments and commonly use theoretically-unfounded communication strategies (e.g. information-giving). It is not known if communication skills teaching during undergraduate training adequately prepares future doctors to engage in effective behaviour change talk with patients. The aim of the study was to examine which behaviour change techniques medical undergraduates use to facilitate lifestyle adjustments in obese patients.

**Methods:**

Forty-eight medical trainees in their clinical years of a UK medical school conducted two simulated consultations each. Both consultations involved an obese patient scenario where weight loss was indicated. Use of simulated patients (SPs) ensured standardisation of key variables (e.g. barriers to behaviour change). Presentation of scenario order was counterbalanced. Following each consultation, students assessed the techniques they perceived themselves to have used. SPs rated the extent to which they intended to make behavioural changes and why. Anonymised transcripts of the audiotaped consultations were coded by independent assessors, blind to student and SP ratings, using a validated behaviour change taxonomy.

**Results:**

Students reported using a wide range of evidence-based techniques. In contrast, codings of observed communication behaviours were limited. SPs behavioural intention varied and a range of helpful elements of student’s communication were revealed.

**Conclusions:**

Current skills-based communication programmes do not adequately prepare future doctors for the growing task of facilitating weight management. Students are able to generalise some communication skills to these encounters, but are over confident and have limited ability to use evidence-based theoretically informed techniques. They recognise this as a learning need. Educators will need to tackle the challenges of integrating theoretically informed and evidence based behaviour change talk within medical training.

## Background

Obesity is a major health concern within developed countries, accounting for a quarter of the UK population (body mass index [BMI] > 30 kg/m^2^) [1]. This has been projected to rise to 60% by 2050 [2]. The increased risk in a range of morbidities and the health benefits of weight loss for obese people are well documented [3-5] and the World Health Organization (WHO) warns of the burden of major chronic diseases that result from obesity [6]. The financial implications of this trend are clear: it is estimated that without action obesity-related diseases will cost the NHS and wider society an extra £49.9 billion per year [2], a situation that is already becoming unsustainable [7]. Medical practice has an important role to play in helping to contain this epidemic and to achieve this, doctors need to be equipped to offer effective weight management. The causes of obesity are complex, multi-dimensional and still poorly understood [8], with genetics, environmental and psychosocial and food consumption factors all contributing [9,10]. Nevertheless, it is recognised that an individual’s lifestyle is a key factor in the development and maintenance of obesity [11]. Unsurprisingly changing the diet and exercise behaviours of susceptible individuals is a top priority for governments [12,13].

Changing behaviour is complex [14]. Nevertheless, the evidence-base for what methods are effective for facilitating behaviour change, particularly in relation to increasing activity and improving diet is vast [e.g. [15]. Behavioural interventions have been shown to be more effective than medical treatments for facilitating weight loss [14]. An important advantage of this evidence-base is that it is informed by theory, drawing on psychological models of why people engage in unhealthy behaviours, and what mechanisms prevent and facilitate behaviour change. Having a theoretical underpinning ensures an understanding of mechanisms and hence predictions can be understood and replicated. Moreover, theoretically-informed communication has been found to be more useful than interpersonal skills [16]. Whilst this literature is substantial and theoretically informed, it is poorly exploited by medical educators, not least because of the range of methodological approaches and specialist behavioural science terminology used. For example, a recent systematic review of undergraduate training in obesity management concluded that medical education was poorly informed by behavioural theories [17].

A further challenge to applying this clinical evidence-base to clinical training is that the techniques and terminology used in the field have been poorly standardized. Consequently, calls have been made for more uniform reporting of clinical interventions [18] and operationalized discrete behaviour change techniques and standardized vocabulary [19], arguing that this will accelerate the progress of intervention adoption. Subsequently Abraham & Michie, compiled a taxonomy of 26 behavioural change techniques (BCT’s) [19], mainly routed in nine different psychological frameworks (e.g. Control Theory [20], Social Cognitive theory [21]). See Table 1 for taxonomy.

**Table 1:** Taxonomy with definitions of 26 behaviour change techniques

**Technique(Theoretical Framework)**	**Definition**
1. Provide information about behaviour health link.	General information about behavioural risk, e.g., susceptibility to poor health outcomes or mortality risk in relation to the behaviour.
2. Provide information on consequences	Information about the benefits and costs of action or inaction, focusing on what will happen if the person does/ does not perform the behaviour.
3 Provide information about others’ approval	Information about what others’ think about the person’s behaviour and whether others will approve or disapprove of any proposed behaviour change.
4. Prompt intention formation	Encouraging the person to decide to act or set a general goal e.g., to make a behavioural resolution such as “I will take more exercise next week”.
5. Prompt barrier identification	Identify barriers to performing the behaviour and plan ways of overcoming them.
6. Provide general encouragement	Praising or rewarding the person for effort or performance without this being contingent on specified behaviours or standards of performance.
7. Set graded tasks	Set easy tasks, and increase difficulty until target behaviour is performed.
8. Provide instruction	Telling the person how to perform a behaviour and/ or preparatory behaviours.
9. Model/ demonstrate the behaviour	An expert shows the person how to correctly perform a behaviour e.g., in class or on video.
10. Prompt specific goal setting	Involves detailed planning of what the person will do including a definition of the behaviour specifying frequency, intensity or duration as well as specification of at least one context, i.e., where, when, how or with whom.
11. Prompt review of behavioural goals	Review and/or reconsideration of previously set goals or intentions.
12. Prompt self-monitoring of behaviour	The person is asked to keep a record of specified behaviour/s (e.g., in a diary).
13. Provide feedback on performance	Providing data about recorded behaviour or evaluating performance in relation to a set standard or others’ performance. Person received feedback.
14. Provide contingent rewards	Praise, encouragement or material rewards that are be explicitly linked to the achievement of specified behaviours.
15. Teach to use prompts/ cues	Teach the person to identify environmental cues which can be used to remind them to perform a behaviour, including times of day, contexts or elements of contexts.
16. Agree behavioural contract	Agreement (e.g., signing) of a contract specifying behaviour to be performed so that there is a written record of the person’s resolution witnessed by another.
17. Prompt practice	Prompt the person to rehearse and repeat the behaviour or preparatory behaviours.
18. Use follow up prompts	Contacting the person again after the main part of the intervention is complete.
19. Provide opportunities for social comparison	Facilitate observation of non-expert others’ performance e.g., in a group class or using video or case study.
20. Plan social support/ social change	Prompting consideration of how others’ could change their behaviour to offer the person help or (instrumental) social support, including “buddy” systems – and/or providing social support.
21. Prompt identification as role model	Indicating how the person may be an example to others and influencing their behaviour or providing an opportunity for the person to set a good example.
22. Prompt self talk	Encourage use self instruction and self encouragement (aloud or silently) to support action.
23. Relapse prevention	Following initial change, help identify situations likely to result in re-adopting risk behaviours or failure to maintain new behaviours and help the person plan to avoid or manage these situations.
24. Stress management	May involve a variety of specific techniques (e.g., progressive relaxation) which do not target the behaviour but seek to reduce anxiety and stress.
25. Motivational interviewing	Prompting the person to provide self-motivating statements and evaluations of their own behaviour to minimize resistance to change.
26. Time management	Helping the person make time for the behaviour (e.g., to fit it into a daily schedule).

Derived from Abraham C, Michie S. A taxonomy of behaviour change techniques used in interventions. *Health Psychol* 2008, 27:379–87.

Sometimes the technique includes an underlying mechanism to facilitate the change that is a common component in more than one theory, for example, *technique 2*: *provide information on consequences*, incorporates themes from the theory of reasoned action [22], the theory of planned behaviour [23], the social cognitive theory and the Information-Motivation-Behavioural Skills model [24], as all these theories outline how consequences of an action may affect or change the attitude towards a target behaviour [19]. This not only provides a strong empirical case for the technique but also highlights the similarities between the different theoretical approaches to behaviour change.

Within routine medical practice however, behaviour change is managed predominantly through information-giving on the risks and consequences of lifestyle habits. The government has invested heavily in information based strategies to help promote healthy lifestyles and behaviours, but information-giving alone has little effect on behaviour despite being the most commonly used technique, especially when considering the complex nature of health behaviours affecting obesity [25].

“*Providing information and persuasive messages can increase people*’*s knowledge of health risks and what action to take*…*is rarely enough on its own*” Department of Health – Choosing Health paper [12]

Health professionals have an important role in encouraging behaviours that promote good health [26]. Three quarters of the UK population visit their GP at least once a year and 90% within 5 years [27], hence primary care practitioners are particularly well situated to engage patients in discussion about health behaviours and weight management. Opportunities to raise behaviour change talk occur commonly in routine consultations, yet are often missed by doctors [28]. In 2002, the Department of Health announced that every opportunity in primary care should be used to promote healthy lifestyles through providing advice on weight, diet and exercise [29]. To achieve this health professionals would need to be skilled in the techniques needed tackle the behaviours associated with weight management.

However, the evidence suggests that health professionals feel ill-equipped and uncomfortable in engaging in these discussions [30,31]. Campbell et al. [32] found that doctors believe they have a positive role to play in weight management but do not know how to treat obesity and underuse effective strategies to alter patients’ lifestyles. Reported barriers that prevent the implementation of behaviour change techniques include pessimism about long-term success, low self efficacy and ambivalence about how best to treat the problem [33]. It has been reported that, doctors from all primary and secondary care perceive their effectiveness at facilitating weight management as poor, have low confidence, feel they have little time within consultations and have inadequate training [30]. Furthermore, they feel weight management is ‘professionally unrewarding’ and is frustrating due to low patient compliance [32]. Faced with a lack of confidence in their ability to effect change and fear of offending patients, doctors commonly prioritise the doctor-patient relationship and avoid behaviour change talk [30]. Evidently doctors do recognise that behaviour change management is integral to medical practice, yet currently feel unable to undertake this role effectively.

That student doctors hold similar prejudices to their professional counterparts is not new: Blumberg & Mellis [34] found that student doctors rated obese individuals much more negatively than average weight persons and described them with adjectives such as ‘unpleasant’, ‘unsuccessful’ and ‘lacking self-control’. Similar attitudes are seen within current cohorts of medical trainees [30]. Training future doctors in obesity lifestyle change management using theoretically underpinned evidence-based techniques could be the key to reducing low self-efficacy and enabling them to deal with such behaviours in their future careers, yet current evidence suggests it is poorly or rarely delivered within medical curricula [17]. In contrast, communication skills training is increasingly a core feature of medical training [35]. It remains unknown if current communication skills training is sufficient to equip students for effectively facilitating behaviour change.

The aims of the study were to examine medical students’ consultations about behaviour change and to identify which theoretically informed behaviour change techniques, if any, they use to facilitate lifestyle change. Specific research questions (the results for which are presented in turn) were:

1. How many behaviour change talk attempts (perceived and observed) do students make in a consultation?

2. What type of behaviour change talk techniques (perceived and observed) do students use in a consultation, and how accurate are students’ estimations?

3. What learning needs do students perceive in behaviour change talk?

4. What are simulated patient views on the motivating things that students say and do?

## Methods

Following approval from a University Senate ethics committee (208/07P), all students in their clinical years (3^rd^, 4^th^ or 5^th,^ approx n=1300), all of who take part in general communication training (4–6 small group sessions per year – experiential patient-centred training with simulated patients, starting with basic consultation skills and moving by 4^th^ year to more challenging scenarios such as breaking bad news) but do not currently receive any behaviour change technique education were invited via a general email announcement to take part in the study. The first 48 students to respond and consent to participate were allocated an appointment to take part in two, ten-minute consultations with a simulated patient. The task for students in each scenario was to facilitate weight loss. As far as possible, scenarios were matched for age, body mass index, health problem that would benefit from weight loss, range of barriers to lifestyle change. The details of each scenario are in below.

Details of scenarios for simulated patients

Scenario A. Mr Adams (age = 53, BMI = 30.5)

· Waiting list for hip replacement for osteoarthritis

· Surgeon advised surgery and recovery will benefit by reducing weight

Scenario B. Mrs Barrowclough (age = 55, BMI = 31.2)

· Borderline Type II diabetes

· GP advised diabetes and further complications could be controlled by reducing weight

The same two simulated patients were used throughout the study which took place over four days. The order that each student encountered the pair of scenarios was counterbalanced to control for any order effects.

Following each consultation, students completed a behaviour change questionnaire. Students were asked to identify techniques they had attempted to use (*perceived behaviour change talk*) from the list of 26 behaviour change techniques [19], and which they would like further training on. Immediately following each consultation, SPs completed a brief questionnaire providing qualitative comments about any aspects of the consultations that might facilitate them to change a health behaviour. They also rated the likelihood of changing their diet or exercise behaviours (on a 5 point Likert scale).

All consultations were audiotaped and transcribed verbatim. Transcripts were subsequently coded using an adaptation of Abraham & Michie taxonomy by one of the authors (LB). Training in the taxonomy took place over several months and coding commenced once good inter-rater reliability was established with the lead authors (unweighted Kappa = .83). For each transcript the number of behaviour change statements was elicited (any attempt to change a diet and/or exercise behaviour). Each of these were then categorised as to the type of technique according to the taxonomy (*actual behaviour change talk*). A copy of the coding manual used is available from the authors.

Data from SP responses to the question “*What was it the student did or said that prompted any new intentions*?” were analysed thematically [36]. Comments were available for all 96 consultations. All comments were initially read and re-read by two of the authors independently and codes identified. Themes were developed using an iterative approach and agreement of the final analysis reached through discussion and search for disconfirmatory evidence within the data corpus [37].

## Results

Of the 48 participants, 28 (52%) were female, age range 20 – 36 yrs (mean = 23.9). Participants were all in the clinical phase of their training in their 3^rd^, 4^th^ or final years (n=21, 20, 7 respectively). No difference was found in behaviour change technique (BCT) statements used between year groups hence data for the year groups are combined (F (2,45)=0.04, NS). 46% of participants were White/Caucasian, 21% were Chinese with the other 33% having a range of ethnicities. Whilst ethnicity is not representative of the cohort, ethnicity was not related to BCT statements used (F (7,40)=1.69, NS).

Students rated their confidence in behaviour change following each consultation (mode = 3, i.e. ‘*slightly confident*’, range 0–5). The mean student confidence level from their 1^st^ consultation was 2.9 and this increased to 3.1 in their 2^nd^ consultation.

1. **How many behaviour change talk attempts do students make in a consultation**?

A total number of 810 behaviour change techniques were made by students (median per student = 16.5, range 9–27). The frequency and types of behaviour change statements was similar for both scenarios (See Table 2) and there were no significant differences found. Table 2 presents data for all participants in frequency form.

2. **What type of behaviour change talk techniques** (**perceived and observed**) **do students use in a consultation**, **and how accurate are students**’ **estimations**?

**Table 2:** Frequency and types of observed behaviour change statements for Scenario A and B

**Behaviour change talk**	**Scenario A**	**Scenario B**
Total no. of statements made	median = 8	median = 8
	(range 4–14)	(range 3–15)
No. statements made about diet	median = 3	median = 3
	(0–10)	(0–10)
No. statements made about exercise	median = 2	median = 3
	(0–6)	(0–8)
No. statements made about both diet and exercise	median = 3	median = 2
	(1–7)	(0–5)
Number of *types* of techniques used	median = 5	median = 5
	(3–10)	(2–9)

Students identified which techniques they had attempted to use, following each consultation. Students reported they used between 1 and 21 different techniques in each consultation. There was no significant difference between the median perceived number of types of techniques used in scenario A was 9 (range = 3–21) and for scenario B was 8 (range = 1–15), Mann U = 242,5, NS.

**Figure 1:**
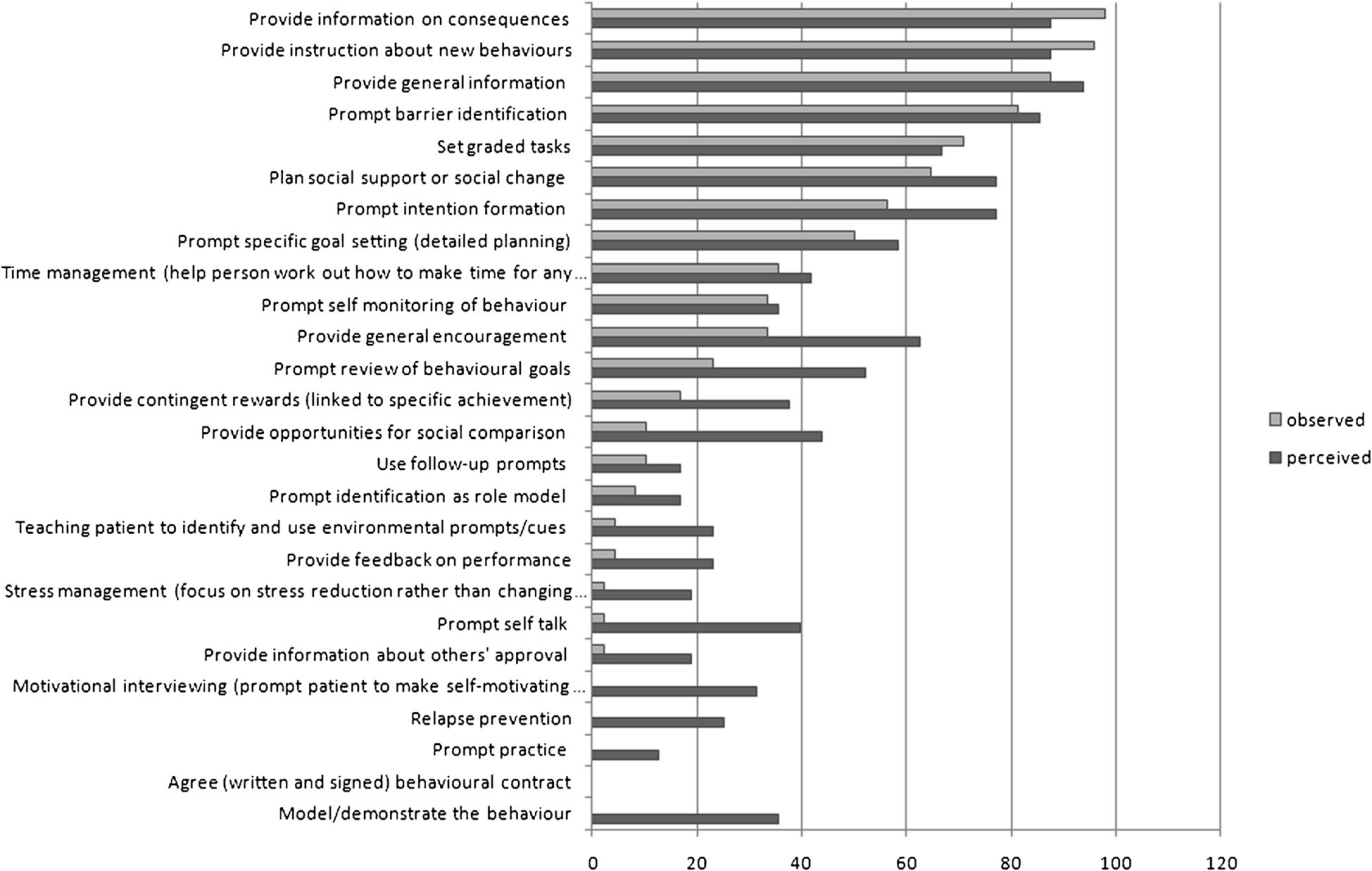
Observed and perceived use of each behaviour change technique.

However, for both scenario A and B, students used significantly fewer types of techniques than they thought they did (scenario A df (degrees of freedom) = 47, t (t-t-test statistic) =6.40, p<.001; scenario B df = 47, t=4.82, p<.001). Figure 1 shows which actual and perceived techniques were used in terms of the percentage of students who used/thought they used each technique. The top 3 techniques for both perceived and observed were the same: *provide information about behaviour*-*health link*, *provide information on consequences* and *provide instruction*. Examples of statements for these techniques are provided in Table 3.

3. **What learning needs do students perceive in behaviour change talk**?

**Table 3:** Examples of behaviour change talk by students (from 3 most commonly used techniques)

**Information on behaviour-health link**	“*All these saturated fats that you*’*re talking about from the deep frying aren*’*t very good*. *They*’*re the ones that clog your vessels up and make you put on weight* – *they are they unhealthy fats*”
**Information on Consequences**	“*Surgery is a lot safer if people keep their weight down*…*carrying extra weight the heart has to pump the blood to extra places*…*heart is working a bit harder already*”
	“*You would get to work feeling extremely refreshed*. *When you do any form of exercise*, *running or whatever*, *it*’*s rewarding*…*you would get to work and think* ‘*I*’*ve done my share of exercise for the day*’”
**Providing Instruction**	“*When you choose your burgers*, *you could choose the healthier ones*, *so the ones with less fat in them if you had a quick look at the box*, *so it means you can still have your burger*, *and hopefully it will still taste as nice and if you grilled it instead then that would be really*, *gradually build up and help*.”

Students were asked to list which techniques they would like more training on. The techniques students wished for more training on were motivational interviewing (72.9% of students), stress management (62.5), relapse prevention (58.3), time management (56.3), and prompting specific goal setting (56.3).

All five of these techniques requested, were used infrequently by students in the study, but many other techniques that were less likely to be identified as techniques they would like more training on (for example, agreeing a behavioural contract), were also used rarely. Additionally, techniques that were used commonly by students were also areas where they would like more training – for example information about the behaviour-health link (37.5%) and information on consequences (45.9%). No technique was requested for training by less than 37.5% of students, suggesting they have a perceived need for training across the range of techniques.

4. What are SP views on the motivating things that students say and do?

SP likelihood to change their behaviour varied considerably across students (scenario A median = 4, range 1 to 5; scenario B median = 3, range 0 to 5). Thematic analysis of SP comments following each consultation revealed a range of features of students’ interactions that they considered would facilitate (or inhibit) them changing their behaviour. Quotes are provided below to support and illustrate the analysis, however all themes were supported by evidence from more than one consultation and from both simulated patients.

### How behaviour change is related to illness outcome

SPs valued learning not only that there was an association between behaviour (e.g. diet) and illness (e.g. diabetes) but also *how* this relationship worked. This on its own was not sufficient to engender intention to change, but did make them open to the student’s ideas.

*Gave me a reasonable understanding of why I needed to lower my glucose levels* &*benefits of that* – *made me slightly more receptive* (ID48)

They also described learning that behaviour change could prevent further symptoms, complications, or the need for medication/treatment and that this was found to be motivating.

‘*The thought that if I do make changes I might avoid medication*’ (ID42)

*The possibility of a complication like a thrombosis on the operating table due to being overweight is an arresting prospect*. *Only a fool would ignore the warning*. (ID43)

It was particularly important that these explanations were presented clearly and in a way that could be understood. Moreover, having a comprehensible model of how the healthier behaviour would affect weight loss was also seen as useful.

*He was good at explaining the whole idea of input and output of energy into the body* (ID80)

### Feeling listened to and understood

SPs valued students who listened to them and made them feel that their situation and concerns had been understood. This could be their thoughts about their illness, life circumstances, or previous experiences in trying to lose weight or attempt a new behaviour.

*Student took the trouble to find out about me and how I felt about my weight*. *Lot of empathy about how I feel disheartened after piling the weight on again after the slimfast fiasco*. *Understood that it made me feel de*-*motivated*…*made me feel hopeful that there were things I could do without going on a drastic diet again*. *I was more willing to listen to ideas because I*’*d been listened to* (ID78)

SPs also described as helpful the occasions when students made efforts to tailor suggestions to their particular circumstances. They reported that this conveyed to them they had been listened to and understood, and felt that the suggestions made under these circumstances were more likely to be useful and practical, and therefore something they might try.

*Student was ready to adapt some of his ideas as to what I should do*, *to what I might be capable of*. (ID52)

Conversely, where students had misunderstood the SP’s circumstances, or ignored a particular concern, this was noted as being demotivating and a reason to inhibit behaviour change.

*I have no interest in jogging so when student offered that as first option it nearly put me off*. (ID74)

Students’ manner and approach to SPs was reported as being highly important in how acceptable the suggestions made were.

*Was very persuasive re walking when she accepted I couldn*’*t jog*. *This could have gone either way with me i*.*e*. *made me more resistant*. *It was her smile and pleasant manner that made me want to at least try it once*, *she really wanted to help*. (ID48)

### Practical, feasible and specific ideas

Although students approach and manner were highly valued, this on its own was insufficient to motivate change: Suggestions needed to be specific and concrete.

*Student was very warm* &*approachable but didn*’*t feel I*’*d got anything specific to try now* (ID44)

The specifics of particular behaviours and how they could be practically achieved were important in SPs’ belief about how likely they would be to implement the suggestions.

*Will think about the food on my plate but need more specific tasks to change*. *Not sure what different proportions* [of food on a plate] *would look like* – *need it to be easier* (ID47)

It was emphasised that to be taken up, any changes needed to be realistic and fit in with their circumstances.

*He did take the time to find out what I actually was doing with my eating habits to give me specific advice that was relevant to me*! *This made changes seem possible without too much* ‘*pain*’. (ID86)

Futhermore, SPs were more likely to consider making changes if the suggestions were viewed as being reasonable and likely to work.

*May consider trying to go for a walk* – *student didn*’*t seem to be expecting me to have to take unreasonably exertive exercise that I*’*m not used to*…*Not sure how long or strong my motivation will be*, *but because I wasn*’*t asked to make drastic changes I might give it a go* (ID60)

A helpful aspect here was where specific goals and plans were made and agreed on. This not only helped SPs create resolve, but also to simply remember the behaviour changes discussed.

*I didn*’*t get anything specific that I could commit to*, *so although part of me felt it was mostly pleasant conversation with a few ideas*, *I would find it difficult to remember too many details*… *I was left feeling we*’*d had a lot of conversation but I can*’*t remember a lot of detail or plans*. (ID88)

### Small changes are worthwhile

SPs reported finding it useful to discover that making small changes were worthwhile. They liked it when students minimised the effort needed to make a difference and felt this was something they would then be willing, at least to try.

*The small change I might consider from this is parking further away at the supermarket* – *that got my attention as something that wouldn*’*t take a lot of effort* (ID49)

In contrast, where the changes suggested were viewed as excessively effortful or strenuous this was given as a reason why they planned not to make any changes.

*Strenuous exercise every night seems a bit much as yet* (ID44)

### Finding the enjoyment in new behaviours

A motivating strategy adopted by one student was to engender the idea that the new, healthier behaviour, could be rewarding in its own right through being enjoyable.

‘*Painted a picture that it could be quite pleasurable* &*I might get used to it being a pleasure rather than a chore* – *was quite enthusiastic about me making a small effort like that*’ (ID43)

SPs described being inspired where efforts were made to find activities or foods that would be enjoyed and ways to maximise the pleasure of the behaviour.

*Suggestion that doing things with partner*’*s support would be a good and doing things together is* ‘*more fun*’ (ID68)

### The value of positive feedback

SPs highlighted that recognition by students of the changes or efforts they had already made was very motivating.

*Gave me encouragement re positive things I*’*d done in monitoring bloods* (ID51)

This included identifying healthy behaviours they were already engaging in and then considering how these could be maximised or developed.

*Encouraged me to think that I perhaps do do a bit of exercise walking around at school* – *perhaps slightly extra walking may be manageable* (ID60)

Moreover, the manner in which some students presented ideas was described as motivating: SPs were more likely to take on the idea of a suggestion when it was presented in an enthusiastic and encouraging manner.

*Student was very encouraging and approachable* &*was able to engage with me so that we both were able to share a laugh at my inability to refuse roly poly pudding*. *Student suggested cutting it in half*, *but understood why I liked them*! (ID79)

A further idea that SPs praised was where students recommended incorporating rewards into their new exercise/diet regimes. This was highly valued, though usually these rewards were food-related treats.

*Giving myself treats e*.*g*. *on a Friday* (*I might break this one and have more often*, *but will probably try*) (ID67)

### Perceiving a non-judgemental stance

SPs commented that the attitudes students conveyed towards them could be encouraging or inhibiting. SPs valued students who didn’t appear to be making judgements about their unhealthy behaviours or the difficulties they had found in making changes. A non-judgemental stance was found to be motivating and encouraged SPs to reduce the barriers they were putting up.

*Student was trying to be non*-*judgemental about the things I was saying which made me feel less obstructive to doing things*. *If we*’*d then gone back to looking more intensively at diet I would*’*ve started to be a little more co*-*operative*. (ID42)

Often however, SPs reported feeling they were being criticised for their behaviours and being told off.

*Student nearly lost me by being a bit directive* ‘*you really must lose weight*, *it*’*s for your own good*!’ *and he said this or similar several times*. *Even though I knew he was right*, *it made me feel negative*…I don’t’ like being made to feel defensive. (ID75)

However it was important that students were honest about what they could offer. This honesty was valued by SPs.

*Admitted he didn*’*t have all the answers but gave some ideas*. *I would take up the offer of seeing a dietician* (ID45)

### Collaborative management

SPs valued it when students communicated that they viewed weight management as a collaborative activity, either by suggesting they engage family members, working with a dietician, or being in a group of others who were trying to lose weight. Most valued was when the collaboration was with the student who offered follow-up appointments to develop the plans and goals and monitor progress/difficulties, and when they presented this as a long term approach they would work on together.

*Looking at things together e*.*g*. *for both diet and exercise*, *student talked about seeing me again in a few weeks as if it were a natural thing that we*’*d keep in touch to see how I was getting on*. (ID63)

### Encouraging patient to take responsibility

However, SPs also described that some students attempted to engage them in making decisions about their commitment to change behaviour and that this could also be very motivating.

*Giving me some credit for being able to think for myself but a plan of asking me back to discuss what i have decided*. *I*’*ll have to have something to tell him when i come back*!!! (ID54)

Through this, students were seen to be empowering them in managing their illness and taking responsibility for their behaviour change and illness management.

*The way the student talked about being able to control diabetes myself* &*not let it control me* (ID59)

## Discussion

To our knowledge, this is the first study to examine medical trainees’ current use of behaviour change talk within the context of weight management. Despite no specific training in behaviour change for obesity, findings revealed that medical undergraduates in the later stages of their training spontaneously made multiple attempts to use theoretically underpinned techniques to change patients’ behaviour. However the techniques used were fewer and less varied than they perceived them to be.

Qualitative research has found that doctors from a wide range of specialties as well as medical trainees are unconfident in behaviour change talk and neglect opportunities to discuss it within clinical settings for fear of damaging the doctor-patient relationship or because they are not aware of effective techniques to use [30]. This is problematic as ‘teachable moments’ occur spontaneously within consultations and doctors who can identify and exploit these are likely to be more successful in facilitating behaviour change in patients [28]. Our study found that within a simulated scenario focused on behaviour change (hence was explicitly a ‘teachable moment’), medical students did attempt behaviour change talk and spontaneously drew on a range of theoretically underpinned behaviour change techniques, that the patients valued, to achieve this. However these attempts were limited and a wide range of techniques were not utilized (although students perceived they were using them). Moreover, many of the techniques identified within the social cognition literature were unfamiliar to students and not used. This suggests there is potential for more focused communication training on behaviour change. Particular techniques will be more appropriate for facilitating change in particular types of health behaviours and training development should include discussion of how to select which approaches to use from the large range of possible techniques.

The study findings support the wider literature that consistently demonstrate that learners overestimate their level of skill and learning needs [38], particularly in areas they are less able in [39]. Consequently students may erroneously consider that their communication skills are sufficient for the task of behaviour change. This is particularly problematic for qualified doctors, who, as life-long learners, become responsible for addressing their own learning needs through self-directed continuing professional training (CPD) [38,40]. Tracey et al. [41] found a poor correlation between doctors’ self-assessment of their knowledge and their subsequent performance in objective tests of their knowledge, and concluded that GPs could not accurately assess their own level of knowledge. Salmon et al. [42] demonstrated that GPs who took part in training opportunities had more positive views of their own efficacy in working with a particular patient group compared with doctors who choose not to take part. They also held more positive views towards these patients compared to their counterparts who declined training for this patient group. This may be particularly important for working with patients with weight problems since there is evidence of negative attitudes towards this patient group, particularly weight bias/stigma [33]. Together the evidence suggests that if CPD is to rely on doctors’ self-perceptions alone these programmes may be seriously flawed [39,43]. A clear way around this issue is to ensure that training occurs at a stage where potential trainees are unable to exclude themselves e.g. undergraduate training, so that at least a basic understanding of effective behaviour change talk techniques is common to all graduates. A recent systematic review found inadequacies in the evidence base of current interventions for training undergraduates in behaviour change for weight management, an evidence-base that included no UK studies [17]: there is clearly a need for further research and practice in this field.

This study is the first to use the behaviour change taxonomy [19] to code verbal consultations. In doing so, we operationalised the framework (which had previously been developed for, and used to code, protocols for interventions). It has been demonstrated that inter-rater reliability can be achieved, and that these theoretically derived techniques can be identified in simulated health professional consultations. Further work is now possible to explore how these techniques are used by more experienced clinicians and during real clinical consultations. Training interventions are often implemented to improve health professionals’ skills in an area without being clear about their baseline skill level. Moreover interventions often only examine students’ confidence or perceived skills as the outcome [17]. The current findings suggest this is unlikely to be an accurate assessment of students’ skills. The current study has also enabled a baseline measure of medical students spontaneous ability in this area, and has allowed investigation of how far generic skills (standard communication skills training) can contribute to specific behaviour change talk ability. Further research is needed to examine the effectiveness of different training interventions and how best these should be delivered.

Using SPs ensured each student has the same ‘patient experience’ and hence the challenges of communicating about behaviour change are standardised across the cohort of students. SPs have been found to be a useful educational tool in learning to manage obesity, leading to increased practitioner confidence and subsequent clinical outcomes for real patients [44]. In this study it means that we can be reasonably confident that each student encountered similar barriers to behaviour change - the aspect of behaviour change that clinicians cite as being particularly challenging [30]. SPs therefore are a useful tool for research and they are routinely used for learning and assessment purposes in medical education [45]. However using actors to play patients potentially introduces artificiality into the study: previous research suggests some students doubt the authenticity of scenario involving SPs, and invest less effort to follow their emotional story when compared to interviewing real patients [46]. This may be a perception, as work has shown that SP consultations are indistinguishable from real patients when used within a clinical setting [47]. Nevertheless, it is possible that within real clinical settings students may have used a different type or number of techniques or invested more greatly in trying to facilitate behaviour change. Qualitative research indicates that within routine consultations doctors neglect opportunities for behaviour change talk, particularly about weight management [30], hence it is suggested that the *amount* of observed behaviour change talk in the current study is likely to be greater than what would be achieved in non-simulated consultations. This is however an empirical question that requires further research.

A further limitation of using SPs is that it was not possible to measure patients’ behavioural (or intended) responses to the consultations. Qualitative comments from simulated patients in this study provided an important adjunct to the quantitative codings as they indicated that many general communication approaches by students were motivating. In particular, SPs valued students who conveyed that they understood the challenges of behaviour change, tailored advice to the individual’s health needs and circumstances, and worked collaboratively to identify practical and graded changes and their illness. Positive feedback and engendering patients with the potential for pleasure in the new behaviour were also highly motivating approaches. However, without obtaining real patient outcome (including any actual behaviour changes) it is not possible to establish if students who used more behaviour change techniques or particular techniques would effect a greater behavioural change in patients. This is an important avenue for future research.

A major limitation of the study is that the ‘opt-in’ recruitment strategy resulted in a low response rate from students: the first 48 students were selected, but it is likely these are not typical of students who responded later or did not respond. Recruitment in medical education studies is often low and concerns have been raised about the bias this introduces to the field [48]. It is highly likely therefore that the sample is not representative of the student population. Students who are most confident in their communication skills may have been more open to putting them under scrutiny. Consequently, the sample is likely to over represent students with greater skill and confidence and who recognise that behaviour change talk is likely to be a useful aspect of their future clinical practice. Therefore, the findings are likely to overestimate students’ level of behaviour change talk skills.

Given the growing obesity epidemic there is clearly a need to ensure behaviour change talk becomes integrated within medical training. Furthermore, this training needs to be informed by evidence-base and theory, which research suggests it currently is not [17]. Historically, behavioural and social sciences have been poorly integrated within medical education [49] and barriers to more effective engagement include lack of staff that are knowledgeable about both behavioural and social sciences *and* medical education, struggles to find space within a crowded curriculum and entrenched epistemologies. However improvements have led to the GMC increasing recognition of the central role of psychology in medical education [50,51] and steps have been taken to develop a behavioural science curriculum that is acceptable to medical educationalists [52]. These developments provide positive platforms for the development of effective training in weight management and behaviour change within undergraduate medical training. Training in this area is not well developed however - Chisholm et al. [17] found that educational interventions were of poor quality and not well evidenced. More recent work in the UK [53] suggests that educators have mixed views about the inclusion of education about weight management. More work needs to be done to ascertain how best teaching can be developed to develop skills and knowledge, and how this translates into clinical practice.

## Conclusions

Current medical education programmes do not adequately equip medical trainees in behaviour change talk. Students may not be able to accurately identify specific techniques they are unskilled at and this may be best delivered during undergraduate training with the support of behavioural and social scientists, with specialist knowledge of theoretical models of how to facilitate behaviour change. Further work is needed to translate the complex theoretically underpinned behaviour change evidence base into accessible techniques that medical educators can use to train the next generation of practitioners.

## Competing interests

The authors declare that they have no competing interests.

## Authors’ contribution

SP and JH designed the study, analysed the data and wrote the paper. LB, HA, SA, PM and CN conducted the study and LB coded the transcripts and conducted inter-rater reliability tests with SP and JH. All authors commented on drafts and approved the final manuscript.

## Pre-publication history

The pre-publication history for this paper can be accessed here:

http://www.biomedcentral.com/1472-6920/13/7/prepub
